# The effects of *Rhizoma Curculiginis *and *Rhizoma Drynariae *extracts on bones

**DOI:** 10.1186/1749-8546-2-13

**Published:** 2007-12-19

**Authors:** Ricky WK Wong, Bakr Rabie, Margareta Bendeus, Urban Hägg

**Affiliations:** 1 Biomedical and Tissue Engineering Research Group, University of Hong Kong, Prince Philip Dental Hospital, 34 Hospital Road, Sai Ying Pun, Hong Kong SAR, China

## Abstract

**Background:**

*Rhizoma Curculiginis *(*Xianmao*) and *Rhizoma Drynariae *(*Gusuibu*) are 'Yang-tonifying' traditional Chinese herbal medicines used to strengthen bones. This investigation aims to assess the systemic effect of extracts of *Rhizoma Curculiginis *and *Rhizoma Drynariae *on bone histomorphology and formation, and their local effect on bone healing.

**Methods:**

For the investigation of the systemic effect, thirty 8-week-old male *BALB/c *mice were divided into three groups: (1) control group, ten mice fed daily with distilled water; (2) *Rhizoma Curculiginis *group, ten mice fed daily with distilled water mixed with *Rhizoma Curculiginis *extract; (3) *Rhizoma Drynarie *group, ten mice fed daily with distilled water mixed with *Rhizoma Drynarie *extract. The mice were fed for five weeks before sacrifice. Twenty micro-tomographic slices with an increment of 0.25 mm were prepared to cover the proximal end of the left tibia of each mouse. Quantitative morphometry of the bone structure was performed. For the investigation of the local effect on bone healing, two bone defects (5 × 10 mm) were created in the parietal bone of each of the three New Zealand white rabbits. Two defects in the first animal were grafted with collagen matrix with *Rhizoma Curculiginis *extract; two defects in the second animal were grafted with collagen matrix with *Rhizoma Drynarie *extract; two defects in the third (control) animal were grafted with collagen matrix alone. The animals were sacrificed on day 14 and the defects were dissected and prepared for histological and ultrastructural assessment.

**Results:**

*Rhizoma Curculiginis *and *Rhizoma Drynariae *extracts altered the bone histomorphology, both increasing the trabecular number by 10% (P = 0.002). *Rhizoma Curculiginis *extract increased bone density by 3.13% (P = 0.122) and *Rhizoma Drynariae *extract increased bone density by 6.45% (P = 0.005). Both *Rhizoma Curculiginis *and *Rhizoma Drynariae *extracts induced new bone formation on the margins of the defects.

**Conclusion:**

Two 'Yang-tonifying' herbs, *Rhizoma Curculiginis *and *Rhizoma Drynariae*, were demonstrated to have systemic effects on bone histomorphology and formation as well as local bone healing.

## Background

For thousands of years, traditional Chinese medicine has been used to treat bone diseases and to promote bone healing [1]. According to Chinese medicine theories, herbs that are 'Yang-tonifying' and act on *Shen *(kidney) strengthen bones. *Shen*, in traditional Chinese medicine, is related to bones [1]. These effects had not been demonstrated by modern scientific methods; thus, we intended to investigate whether the crude extracts of these herbs can indeed increase bone formation. New leads of therapeutic chemicals may provide possibilities for treating osteoporosis, reduce bone resorption after bone grafting surgery or promote bone healing after bone surgery or fracture.

One of the 'Yang-tonifying' herbs for strengthening bones is *Rhizoma Curculiginis *(*Xianmao*), the dried rhizome of *Curculigo orchioides *Gaertn. (Amaryllidaceae) [2]. The 'Yang-tonifying' effect of *Rhizoma Curculiginis *is hormone-like. Studies show that oral administration of 10 g/kg of the decoction of *Rhizoma Curculiginis *significantly increased the weight of the lobus anterior hypophysis, ovary and uterus in rats. *Rhizoma Curculiginis *can potentiate the luteotrophic activity of the hypothalamus-pituitary-ovary system [3]. In traditional Chinese medicine, *Rhizoma Curculiginis *is used to reinforce the Yang in *Shen *to treat impotence, limpness of the limbs, arthritis of the lumbar and knee joints, and to strengthen tendons and bones [2]. Current research mainly focuses on identifying the chemical components of this herb [4,5]. Thirteen cycloartane triterpene saponins named curculigosaponins A to M were isolated and identified in *Rhizoma Curculiginis *[6,7]. Two other triterpenes, curculigol and 31-methyl-3-oxo-20-ursen-28-oic acid, were also isolated from *Rhizoma Curculiginis *[8,9]. Moreover, phenyl glycosides curculigoside B, curculigines B and C, and an aliphatic compound 25-hydroxy-33-methylpentatricontan-6-one were identified in *Rhizoma Curculiginis *[7,9].

*Rhizoma Drynariae *(*Gusuibu*), the dried rhizome of perennial pteridophyte *Drynaria fortunei *(Kunze) J. Sm. (Polypodiaceae), is another 'Yang-tonifying' herb for treating bone diseases [2]. According to traditional Chinese medicine theories, *Rhizoma Drynariae *acts on *Shen *and *Gan *(liver) and is used for replenishing *Shen*, treating deficiency syndrome of the kidney and promoting the healing of fracture and relieving pain; it is also used in treating traumatic injuries and bone fracture [2]. The extract of *Rhizoma Drynariae *which contains flavonoid and triterpenoid compounds was shown to increase bone cell viability, intracellular total proteins, alkaline phosphatase and acid phosphatase [10]. Naringin, a major flavonoid component, went up by 1%. The triterpenes isolated from *Rhizoma Drynariae *include 24-ethyl-9, 19-cyclolanost-25-en-3; 3-ol, hop-22(29)-ene and fern-9(11)-ene [2]. Our laboratory recently demonstrated that naringin, a flavonoid component increased new bone formation locally [11].

Micro-computed tomography (micro-CT) permits non-invasive imaging and quantitative morphometry of the bone structure in two and three dimensions [12,13]. For visualizing trabecular architecture, micro-CT is better than conventional methods of histological sectioning, e.g. planar radiography and medical computed tomography [14]. Micro-CT can produce images with resolution in tens of microns to show micro-architecture of bone and bone grafts [15,16]. Micro-CT was commonly used for studying the effects of different agents on bone histomorphology [17,18]. By using mice as an animal model [19], it is possible to compare the systemic effect of different agents on bones with normal mice as control. However, for studying the local effect of different agents on the healing of bone defects, it is necessary to use other animal models (e.g. rabbits) that allow histological examination of bones and other tissues surrounding the agents and to utilize a carrier that allows the release of these agents. The present study aims to investigate the systemic effect of crude extracts of *Rhizoma Curculiginis *and *Rhizoma Drynariae *on bone histomorphology in normal mice using micro-CT and the local effect of these extracts on a bone defect in rabbits.

## Methods

### Identification and preparation of *Rhizoma Curculiginis *and *Rhizoma Drynariae *extracts

*Rhizoma Curculiginis *and *Rhizoma Drynariae *were purchased in a local Chinese medicine store and were identified morphologically, histologically and chemically according to standard Chinese herbal identification procedures [20,21]. Initially, the morphology and histology of the herbs were compared with standard photographs obtained from the School of Traditional Chinese Medicine, the University of Hong Kong. The actual identification procedures were performed by the authors in the Hard Tissue Laboratory, the University of Hong Kong. Thin layer chromatography was used to separate the components of *Rhizoma Curculiginis *ethanolic extract. Potassium ferrocyanide (2%) and ferric chloride were added to the extract, which produced bluish spots (compared with the standards), an indication of the authenticity of the herb [21]. Methanolic extract of *Rhizoma Drynariae *was used for thin layer chromatography. The extract was separated by benzene-methanol-butanone (at a ratio of 3:1:1) and tested with ferric chloride-ethanol solution (1%). Brownish colored extract was compared with naringin standard [20]. A sample of each herb was stored in the Hard Tissue Laboratory. *Rhizoma Curculiginis *and *Rhizoma Drynariae *extracts were prepared according to the protocol for commercial production of injection preparation of traditional Chinese medicine in China [22]. For every 4 g of *Rhizoma Curculiginis *or *Rhizoma Drynariae *powders, 40 ml of distilled water was added and the mixtures were boiled with stirring on a hot plate for 4 hours. Distilled water was added occasionally to prevent the mixtures from drying. The final volume of the mixtures was made up to 4 ml by adding distilled water. The mixtures were cooled to room temperature and then centrifuged. The supernatants were collected and filtered with a 0.22 μm sterile syringe filter into a sterile glass bottle. The extracts contained 1 g/ml of *Rhizoma Curculiginis *or *Rhizoma Drynariae*. This method of extraction is widely used for obtaining water soluble fractions in Chinese medicinal herbs [23].

### Investigation of systemic effect of *Rhizoma Curculiginis *or *Rhizoma Drynariae *extracts

All animals were obtained from the Laboratory Animal Unit at the University of Hong Kong where they were kept under standard conditions. The temperature was kept between 22°C and 24°C. The light cycle was from 8 am to 8 pm daily. Each animal was kept individually in a cage and fed with standard diet in the Laboratory Animal Unit. The animal handling and experimental protocol was approved by the Committee for the Use of Living Animals in Teaching and Research, the University of Hong Kong.

The mice were bred by the Laboratory Animal Unit, the University of Hong Kong. The doses of both *Rhizoma Curculiginis *and *Rhizoma Drynariae *for human were 0.2 g/kg/day as suggested in the Chinese Pharmacopeia [2]. The doses of the herbs for mice were estimated in a pilot study. Thirty 8-week-old male *BALB/c *mice were divided into three groups as follows:

1. Control group (C_mice_): Ten *BALB/c *mice fed with normal diet and distilled water.

*2. Rhizoma Curculiginis *group (XM_mice_): Ten *BALB/c *mice fed with normal diet and distilled water mixed with *Rhizoma Curculiginis *extract (0.5 g *Rhizoma Curculiginis *in 250 ml of water).

*3. Rhizoma Drynariae *group (G_mice_): Ten *BALB/c *mice fed with normal diet and distilled water mixed with *Rhizoma Drynariae *extract (0.5 g *Rhizoma Drynariae *in 250 ml of water).

The mice were kept individually (one animal per cage) for five weeks before sacrificed. The drinking solutions, which did not contain any suspending herbal particulates, were freshly prepared every day.

The bone samples (proximal tibia) were carefully dissected after sacrifice of the mice. The sample was then fixed with buffered saline and placed onto a sample holder. The proximal tibia is a long bone which can be easily inserted in the micro-CT chamber, allowing easy standardization in specimen positioning. These procedures were originated from other similar micro-CT studies [18]. The investigator was blinded to the treatment of each mouse. The morphometric parameters were determined automatically by computer without human interference. The bone samples were scanned through 360° by a compact fan-beam-type tomography instrument μCT20 (Scanco Medical AG, Bassersdorf, Switzerland). Samples were placed in an air-tight cylindrical sample holder filled with formaldehyde to preserve the sample for the duration of the measurement. The sample holder was marked with an axial alignment line on the outside of the tube to allow consistent positioning of the specimens within the holder. By connecting the alignment notch to the sample holder with the μCT20 turnable, precise positioning of the bone within seconds was achievable. A typical analysis consisted of a scout view to ensure accurate and consistent positioning of slides, selection of the examination volume, automatic positioning, measurement, offline reconstruction and evaluation. Scout (enlarged) views were taken to ensure the precision of finding the anatomic location in each bone, a sample size estimated from similar studies was selected to minimize the effect of the location variation.

**Figure 1:**
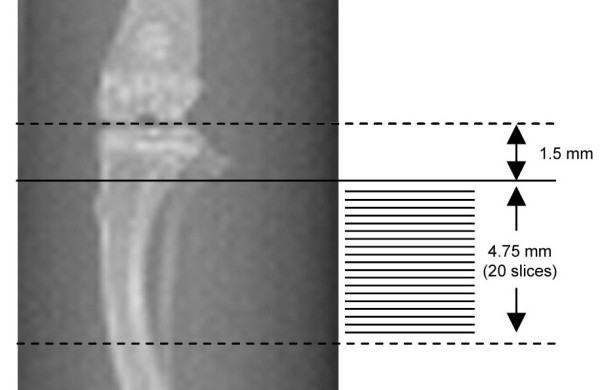
Scout view (× 3) of tibia (left). The proximal end of the tibia was covered by the 20 sections. The first, most proximal slice was 1.5 mm away from the proximal end of the tibia. Sample of micro-CT scan section (right). The fibula was excluded during analysis (× 20).

Modified protocols from Zhang *et al. *[24] and Ishimi *et al. *[18] were followed: twenty micro-CT slices/sections (0.25 mm apart) were taken to cover the proximal end of the left tibia. The most proximal slice was 1.5 mm away from the proximal end to avoid possible morphological variation in the proximal head (Figure 1). Quantitative morphometry of the bone structure was performed with the μCT20 computer system. The fibula was excluded from measurement. The micro-CT reconstruction parameters were as follows: sigma: 1.2; support: 2; threshold: 140; increment/scan thickness: 0.25 mm; resolution: high (1024 × 1024 pixels).

Data were analyzed using statistical analysis software Graphpad Instat (v.2.04a). The arithmetic mean and standard deviation (SD) were calculated for each group. The means (XM_mice _and C_mice_; G_mice _and C_mice_) were compared by the Welch's unpaired t test which does not assume equal variances, with P < 0.05 chosen as the critical level of statistical significance.

### Investigation of local effect of *Rhizoma Curculiginis *or *Rhizoma Drynariae *extracts

The methodology and animal model used in the current study have been described previously [11]. Six 10 × 5 mm^2 ^full-thickness bone defects were created in the parietal bones of three inbred New Zealand white rabbits. The rabbits were five months old (adult stage) and weighed between 3.5 kg and 4.0 kg. The animal handling and experimental protocol was approved by the Committee on the Use of Live Animals in Teaching and Research, the University of Hong Kong. In the experiment, two defects in the first animal were grafted with collagen matrix with *Rhizoma Curculiginis *extract; two defects in the second animal were grafted with collagen matrix with *Rhizoma Drynarie *extract; two defects in the third (control) animal were grafted with collagen matrix alone. The animals were pre-medicated one hour before surgery with oxytetracycline hydrochloride (200 mg/ml, 30 mg/kg body weight) and buprenorphine hydrochloride (0.3 ml/kg body weight), supplemented with diazepam (5 mg/ml, 1 mg/kg body weight). In order to maintain the level of neuroleptanalgesia, increments of Hypnorm (0.1 ml/kg) were given at 30-minute intervals during the operation.

The surgical procedure consisted of the creation of two 10 × 5 mm full-thickness (approximately 2 mm) cranial defects, devoid of periosteum, using templates, in the parietal bones. The defects were produced using round stainless steel burs (1 mm in diameter) on a low speed dental drill. Outlines of the defects were made initially by making holes of full thickness the parietal bone using a stainless steel wire template bent to the required size of the defect. The holes were joined to complete the process. During the cutting of the bones, copious amount of sterile saline was used for irrigation and to minimize thermal damage to the tissues. In the first experimental animal, the defects were filled with purified absorbable fibrillar collagen matrix (Collagen Matrix, NJ, USA) with 0.2 ml 1 g/ml *Rhizoma Curculiginis *extract. In the second experimental animal, the defects were filled with the same collagen matrix with 0.2 ml 1 g/ml *Rhizoma Drynarie *extract. The grafts were prepared 15 minutes before grafting. In the control animal, the defects were grafted with 0.02 g of the same collagen matrix mixed with 0.2 ml water for injection.

All wounds were closed with interrupted 3/0 black silk sutures. No attempt was made to approximate the periosteum to prevent the barrier effect. Postoperatively, the rabbits were given oxytetracycline hydrochloride daily for ten days and buprenorphine hydrochloride for two weeks. The animals were monitored under a standardized protocol during postoperative period with the supervision of the veterinary surgeon for any unhealthy signs or side effects. Medication for the animals during this period was as follows: 30 mg/kg of oxytetracycline hydrochloride intramuscular injection every 30 minutes; 50 μg/kg of Temgesic subcutaneous injection daily (for two weeks); 60 ml of saline and 10 ml Dextran 40 (10% dextrose solution in 0.9% saline solution) subcutaneous injection daily until appetite recovered; 10 ml of Dextran 40 in 350 ml drinking water daily until appetite recovered; vitamin B1, B6 and B12.

Two weeks after surgery, the animals were sacrificed with sodium pentobarbitone. Immediately after death, defects and surrounding tissues were removed for histological preparation.

Tissues were fixed in 10% buffered saline solution, demineralized with K's Decal Fluid (sodium formate/formic acid) and double embedded in celloidin-paraffin wax. Serial, 5-μm-thick sections of the whole defect were cut perpendicular to the long axis. The slides were stained with Periodic acid-Schiff stain which allowed easy identification of new bone formation.

For the investigation of the ultra-structure of new bone formation, some tissues of the animal grafted with *Rhizoma Drynarie *extract was fixed with Karnovsky solution, decalcified in K's Decal Fluid and double embedded in celloidin-paraffin wax. The specimens were cut into 3-μm-thick serial sections, perpendicular to the long axis, and stained with hematoxylin and eosin. Histological sections were used to locate the area of the interface between the graft and the host bone on the tissue blocks. The areas of interest were then fixed, post-fixed in osmium tetroxide (OsO_4_) and trimmed into 1 mm blocks and embedded in resin. Semi-thin sections from resin block were sectioned and stained with toluidine blue for further orientation and reduction of the block face. Ultra-thin sections (90 nm thickness) were cut with a diamond knife to and mounted on metal grids (200 meshes). Sections were then stained with uranyl acetate and lead citrate, and examined under a transmission electron microscope (EM208S, Phillips Electron Optics BV, Netherlands).

## Results

### Investigation of systemic effect of *Rhizoma Curculiginis *or *Rhizoma Drynariae *extracts

All animals remained in excellent health throughout the course of the experiment. There was no evidence of side effects in any of the animals. The mean (SD) weight of the mice was 28.46 (1.03) g and there was no significant difference in weight between the experimental and control groups before and after the experiment. The mean (SD) daily water consumption of each mouse was 6.6 ml. There was no significant difference in daily water consumption between the XM_mice_: 6.7 (1.1) ml, G_mice_: 6.5 (1.1) ml and C_mice _groups: 6.6 (1.3) ml.

Micro-CT scanning was performed on the proximal tibia of the XM_mice_, G_mice _and C_mice _groups. 2D-histomorphometry was calculated from the micro-CT system. Compared with the C_mice _control group, trabecular number increased 10.00%; trabecular thickness and trabecular separation decreased 7.01% and 9.66% respectively in the XM_mice _group. The low trabecular separation is consistent with the high trabecular number, i.e. finer trabecular pattern with more and thinner trabeculae. In the XM_mice _group, the low trabecular separation is also consistent with increased bone surface to tissue volume and bone surface to bone volume ratios which went up 10.03% and 6.68% respectively. In the G_mice _group, compared with the C_mice _control group, trabecular number increased 10.00%; trabecular thickness and trabecular separation decreased 3.78% and 9.66% respectively. *Rhizoma Drynariae *extract caused a lower trabecular thickness, which was consistent with the overall increase in the bone volume/tissue volume ratio (up 6.45%), an indicator of bone density (Table 1).

**Table 1:** Micro-CT scan and 2D-morphometry

Parameter	C_mice_	XM_mice_		G_mice_	
	Mean (SD)	Mean (SD)	% diff (P-value)	Mean (SD)	% diff (P-value)
Bone volume/tissue volume (%)	17.748 (1.073)	18.304 (1.096)	3.13%↑ (0.122)	18.892 (1.207)	6.45%↑ (0.005*)
Trabecular number (1/mm)	0.781 (0.080)	0.860 (0.049)	10.00%↑ (0.002*)	0.859 (0.054)	10.00%↑ (0.002*)
Trabecular thickness (mm)	0.291 (0.022)	0.271 (0.010)	7.01%↓ (0.002*)	0.280 (0.013)	3.78%↓ (0.069)
Trabecular separation (mm)	0.321 (0.036)	0.290 (0.020)	9.66%↓ (0.002*)	0.290 (0.020)	9.66%↓ (0.002*)
Bone surface/bone tissue volume (1/mm)	2.454 (0.246)	2.700 (0.156)	10.03%↑ (0.002*)	2.699 (0.165)	10.00%↑ (0.002*)
Bone surface/bone volume (1/mm)	14.067 (1.122)	15.006 (0.644)	6.68%↑ (0.005*)	14.625 (0.653)	4.00%↑ (0.070)

C_mice_: control group

XM_mice_:*Rhizoma Curculiginis *group

G_mice_: *Rhizoma Drynariae *group

↑:increase

↓:decrease

SD: standard deviation

% diff: % difference

*: P < 0.05

For method error analysis, readings of ten sections recorded at two separate micro-CT scanning sessions by the same examiner were randomly drawn and the readings were then compared. The method error of the image analysis (∑d22n
 MathType@MTEF@5@5@+=feaagaart1ev2aaatCvAUfKttLearuWrP9MDH5MBPbIqV92AaeXatLxBI9gBaebbnrfifHhDYfgasaacPC6xNi=xH8viVGI8Gi=hEeeu0xXdbba9frFj0xb9qqpG0dXdb9aspeI8k8fiI+fsY=rqGqVepae9pg0db9vqaiVgFr0xfr=xfr=xc9adbaqaaeGacaGaaiaabeqaaeqabiWaaaGcbaqcfa4aaOaaaeaadaWcaaqaamaaqaeabaGaemizaq2aaWbaaeqabaGaeGOmaidaaaqabeqacqGHris5aaqaaiabikdaYiabd6gaUbaaaeqaaaaa@3339@) is 0.003194 mm^2^, where *d *is the difference between the two readings of a pair and *n *is the number of double readings. The error was insignificant compared with the results. The duplicate intra-observer registrations of the 10 randomly drawn sections were also insignificant (P = 0.1679).

### Investigation of local effect of *Rhizoma Curculiginis *or *Rhizoma Drynariae *extracts

All animals remained in excellent health throughout the course of the experiment and recovered rapidly after operation. There was no evidence of side effects or infection in any of the animals.

**Figure 2:**
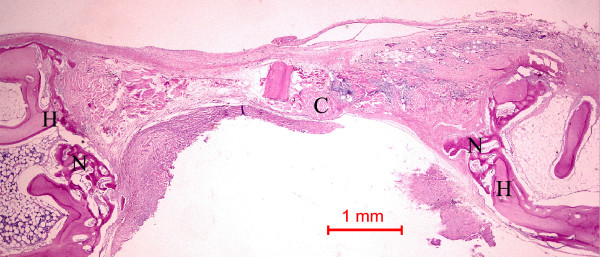
Photomicrograph of bone defect grafted with *Rhizoma Curculiginis *extract in collagen matrix on day 14. New bone (N) can be seen spanning the defect. H: Host bone. Some collagen matrix (C) remained at the center of the bone defect (Periodic acid-Schiff stain; original magnification × 40).

**Figure 3:**
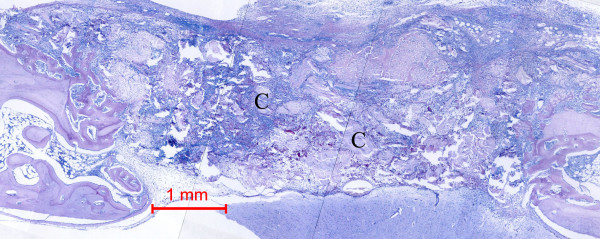
Photomicrograph of bone defect grafted with *Rhizoma Drynariae *extract in collagen matrix on day 14. New bone can be seen spanning the defect. Some collagen matrix (C) remained at the center of the bone defect (Periodic acid-Schiff stain; original magnification × 40).

In the rabbits grafted with *Rhizoma Curculiginis *or *Rhizoma Drynariae *extracts in collagen matrix, new bone was formed at the host bone – graft interface tended to grow across the defect (Figures 2 and 3). Integration of the *Rhizoma Curculiginis *or *Rhizoma Drynariae *extracts and collagen with the recipient bed was characterized by the appearance of new bone. No cartilage was found. At higher magnification, new bone could be seen growing around a collagen matrix fragment and tended to grow towards and amalgamate with the collagen matrix. The presence of bone cells indicated that new bone was formed.

**Figure 4:**
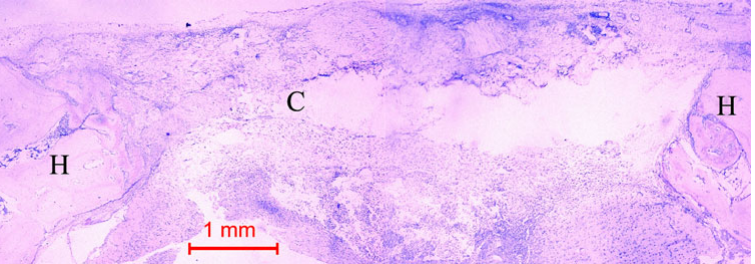
Photomicrograph of bone defect grafted with collagen matrix (positive control) on day 14. No bone could be seen across the defect except a little new bone near the ends of the host bone (H). Collagen matrix (C) remained across the bone defect (Periodic acid-Schiff stain, original magnification × 40).

In the control rabbit, little new bone was formed at the host bone/graft interface. Some collagen fibers were present at the center of the defects (Figure 4). In all rabbits, the defects were healed, with fibrous tissue bridging across the defects.

**Figure 5:**
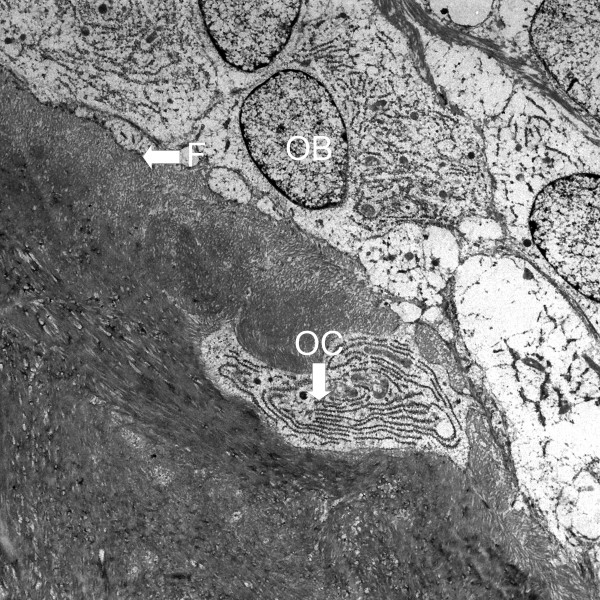
Transmission electron micrographs show the area near the newly formed bone of bone defect grafted with *Rhizoma Drynariae *extract in collagen matrix. (F: mineralization front; OB: osteoblasts; OC: osteocytes; original magnification × 3500).

The area of interest for ultra-structural study was taken near the newly formed bone and its surrounding connective tissue. At the mineralization front (F), osteoblasts (OB) with abundant rough ER and newly entrapped osteocytes (OC) could be seen (Figure 5).

## Discussion

According to traditional Chinese medicine, the herbs that affect *Shen *have effects on bones [2]. Two Shen-affecting herbs, namely *Rhizoma Curculiginis *and *Rhizoma Drynariae*, were used to test this hypothesis. The non-destructive and precise micro-tomography system for measuring static morphometrical and architectural parameters of bones [14] showed systemic effects of *Rhizoma Curculiginis *and *Rhizoma Drynariae *extracts on bones.

The administration of *Rhizoma Curculiginis *extract for five weeks changed the trabecular pattern of the long bone in mice. The increase in bone density, indicated by the bone volume/tissue volume ratio, was not statistically significant. Further research is needed to investigate whether the bone remodeling rate is increased by the administration of *Rhizoma Curculiginis *extract and what biological mechanisms are.

This study showed that both *Rhizoma Curculiginis *and *Rhizoma Drynariae *altered bone histomorphological pattern and that *Rhizoma Drynariae *increased bone density. In addition, both of these herbal extracts induced new bone formation on the margins of the bone defects, whereas little new bone formation was seen in the control groups. The ultra-structural study of the new bone formed in the rabbit with *Rhizoma Drynariae *extract was to investigate the structure of the new bone. The identification of osteoblasts and osteocytes in the newly formed bone showed that the newly formed hard tissue was actually bone instead of calcification of the collagen fibers.

While this study aims to identify the effect of crude extracts of *Rhizoma Curculiginis *and *Rhizoma Drynariae *on bones, we can speculate the action mechanism of the effect. The bone anabolic ability of *Rhizoma Drynariae *shown in this study is likely to be related to angiogenesis and/or osteogenesis through the up-regulation of the expression of osteogenic factors. The mechanism of the osteogenic effect of *Rhizoma Drynariae *can be partially explained by the activation of BMP-2 gene expression through the HMG-CoA reductase inhibition effect of naringin. Further studies are required to investigate whether other active components of *Rhizoma Drynariae *are also osteogenic. In *Rhizoma Curculiginis*, a new orcinol glucoside that has potent antioxidative activities similar to that of naringin was identified [5]. It is possible that orcinol glucoside also affects the redox reaction of the HMG-CoA transformation to mevalonate and triggers the BMP-2 gene expression. Ko and Leung [25] recently reported that 'Yang-tonifying' herbs enhance ATP generation capacity in mitochondria and exhibit antioxidant effect. Whether the APT generation and antioxidant effect are related to the osteogenic effect is of interest in our future studies. It is well known that the effect of any compound on bones is dependent on the site of the bone. As femur neck and lumbar are most susceptible to fracture, future studies of the effects of *Rhizoma Curculiginis *and *Rhizoma Drynariae *on femur neck and lumber are also warranted.

## Conclusion

Two 'Yang-tonifying' herbs, *Rhizoma Curculiginis *and *Rhizoma Drynariae*, were demonstrated to have systemic effects on bone histomorphology and formation as well as local bone healing.

## Competing interests

The author(s) declare that they have no competing interests.

## Authors' contributions

RW conceived the research design. RW, AR, MB and UH contributed to the research work, supervision of the technicians, and drafting of the manuscript. All authors read and approved the final manuscript.
